# Analysis of genomic variation in lung adenocarcinoma patients revealed the critical role of PI3K complex

**DOI:** 10.7717/peerj.3216

**Published:** 2017-05-10

**Authors:** Zhao min Deng, Lin Liu, Wen hai Qiu, Yong qun Zhang, Hong yan Zhong, Ping Liao, Yun hong Wu

**Affiliations:** 1 Hospital of Chengdu Office of People’s Government of Tibetan Autonomous Region, Chengdu, China; 2 West China Second University Hospital, Chengdu, China

**Keywords:** Lung adenocarcinoma, Genomic variation, CNV, Mutations, PI3K

## Abstract

**Background:**

Molecularly targeted therapies improved survival status of some patients with lung adenocarcinoma, which accounts for 40% of all lung cancers, and in-depth study of gene alterations is important for the personalized treatment.

**Methods:**

The legacy archive data of clinical information and genomic variations under the project TCGA Lung Adenocarcinoma were downloaded from the GDC Data Portal using R package TCGAbiolinks. The significantly aberrant copy number variants segments were figured out using GAIA. After annotation, the genes involving CNV were used to get enriched pathways. Recurrent amplifications and deletions were identified and visualized by OncoPrint. Genomic alterations in cancer, including CNV and mutations, were represented in Circos.

**Results:**

The significantly aberrant CNV segments were found, and the genes involved were associated with the immune system. In an analysis of 517 mutation annotated files, we highlighted 63 highly recurrent mutated genes which were associated with lung cancer signaling. These genes involved in important pathways related to cancer progression. The intersections between the genes involving in the significantly aberrant CNV and the genes harboring recurrent somatic SNP were extracted. The PI3K protein family acted as critical roles in the lung adenocarcinoma, since the components of the PI3K protein family include PIK3C2B, PIK3CA, PIK3R1 and so forth were presented in the intersections.

**Conclusion:**

We represented a comprehensive annotation of genomic alterations in lung adenocarcinoma and proposed that PI3K signaling proteins were critical for it.

## Introduction

Lung cancer is one of the deadliest cancers worldwide, causing about 1.59 million deaths every year, about 40% of which are led by lung adenocarcinomas (Stewart & Wild, 2016). Since lung adenocarcinoma are diagnosed, in most times, as locally advanced or metastatic status, the five-year survival rate of patients with lung adenocarcinoma is only about 15%, even though molecular diagnosis and targeted medicines had been utilized (Imielinski et al., 2012).

The current clinical staging system is usually utilized as the standard for predicting prognoses, and the surgical resection is typically regarded as standard treatment for adenocarcinoma patients. However, close to 35% of surgically treated stage I patients finally encounter relapse after the initial surgery, suggesting a subgroup of patients diagnosed as early stage had residual cancer cells undetectable by current techniques (Tomida et al., 2009). Recently, molecularly targeted therapies have dramatically improved the survival status for patients with mutant EGFR1 or translocated ALK, RET, or ROS1 (Cancer Genome Atlas Research , 2014). Mutant BRAF and ERBB2 (Stephens et al., 2004) are also found to be target candidates. However, most lung adenocarcinomas either lack known driver oncogenes, so most patients still treated with conventional chemotherapy. Therefore, knowledge of additional genes altered in lung adenocarcinoma is needed for personalized therapy.

In this study, we analyzed the results of the genotyping array and whole exome sequencing (WXS) on lung adenocarcinoma from TCGA to figure out the copy number variations (CNV), the single-nucleotide polymorphism (SNP) and indels involving in lung adenocarcinoma initiation and progression. We identified both CNVs and mutations on PI3K protein complexes, which indicated their critical roles in the lung adenocarcinoma. Though Class 1A of PI3K complexes had been proven to participate in the cancer pathway, there was limited attention on the Class IB and Class II. These results represent a comprehensive annotation of somatic alterations and CNVs in lung adenocarcinoma and also propose a direction of PI3K class proteins.

## Methods

### Data

The legacy clinical information and genomic variations of TCGA Lung Adenocarcinoma were available in Genomic Data Commons (GDC) Data Portal. The level-3 CNV data were directly /downloaded as a delimiter table and the mutations were organized as MAF files (Colaprico et al., 2016). The retrieved genomic alteration data, which were shown as legacy archive, were processed by TCGA using the reference of hg19. We analyzed CNV data generated by tumor and matched normal material from 550 lung adenocarcinoma patients and SNP data from tumor tissues from 399 patients (Table 1). The major histologic types of lung adenocarcinoma involved included lung papillary adenocarcinoma, lung bronchioloalveolar carcinoma nonmucinous, mixed subtype lung adenocarcinoma, and so on.

**Table 1:** Datasets statistics. (A) Clinical features and (B) selected sequencing statistics for 522 lung adenocarcinoma cases.

Category	Number
(A)	
Age at diagnosis (median; range)	66 (33–88)
Gender	
Male	242
Female	280
Number of pack per year smoked	44 (0–154)
Tumor stage	
I	279
II	124
III	85
IV	26
NA	8
(B)	
Total Patients	522
Patients with CNV data	
Tumor samples	518
Normal tissues of patients	421
Patients with SNP data	517

### Identification of recurrent CNV

The CNV dataset came from platform Affymetrix Genome-Wide Human SNP Array 6.0 in TCGA. The level 3 data for both primary solid tumor samples and paired normal tissues were queried using TCGAbiolinks (Colaprico et al., 2016). Data for all the samples were downloaded and prepared into the format of SummarizedExperiment (Huber et al., 2015).

Genomic Analysis of Important Aberrations (GAIA), an iterative procedure where a statistical hypothesis framework is extended to take into account within-sample homogeneity, was used to figure out the most significant recurrent CNV (Morganella, 2010). GAIA used a conservative permutation test to calculate the probability distribution of the contemporary mutations expected for non-driver markers. Afterwards, the statistical significance of each marker was calculated based on the observed data. Finally an iterative procedure was used to identify the most significant independent regions which were supposed to be driver mutations. Furthermore, GAIA requires genomic probes metadata, which are available from the FTP site (ftp://ftp.broadinstitute.org/pub/GISTIC2.0/hg19_support/) of Broad Institute (Mermel et al., 2011). FDR was counted to identify significant CNV segments using R package qvalue (Storey et al., 2015).

The aberrant recurrent genomic regions in cancer, as identified by GAIA, were annotated to figure out the genes that were significantly amplified or deleted. Using biomaRt (Durinck et al., 2009), the genomic ranges of all human genes were obtained and the full length genes that were located within significant aberrant regions were extracted. The genes that were significantly amplified or deleted were used to carry out the pathway enrichment using DAVID Bioinformatics Resources (Huang, Sherman & Lempicki, 2009a; Huang, Sherman & Lempicki, 2009b) which suggested their biological functions.

### Identification of recurrent SNP and Indels

The Mutation Annotation Format (MAF) files, which contained somatic or germline mutations with validated or putative state generating from whole exome sequencing (WXS), were downloaded using TCGAbiolinks (Colaprico et al., 2016). This package also summarized all the pathways from KEGG, Reactome or other databases and we extracted all the pathways relating with lung cancer including small cell lung cancer signaling and non-small cell lung cancer signaling. Finally, we filtered the genes with mutations involving the lung cancer-related pathways.

Recurrent amplifications and deletions were identified and visualized by OncoPrint, which is compact means of visualizing distinct genomic alterations, including somatic mutations and CNV across a set of cases (Gao et al., 2013). Individual genes are represented as rows, and individual cases or patients are represented as columns. In order to visualize multiple genomic alteration events by OncoPrint plot, we utilized R package complexHeatmap (Gu, 2016). We defined SNPs as blue, insertions as green and deletions as red. The upper barplot indicates the number of genetic mutation per patient, while the right barplot shows the number of genetic mutations per gene. The grades of lung adenocarcinoma and the history of smoking were added as annotations for the patients.

### Combination of CNV and mutations

Genomic alterations in cancer, including CNV and mutations, were represented in an effective overview plot named Circos (Krzywinski et al., 2009). R package circlize was used to represent significant CNV from GAIA and recurrent mutations (Gu et al., 2014).

## Results

### Genes deleted were involved in immunity

**Figure 1:**
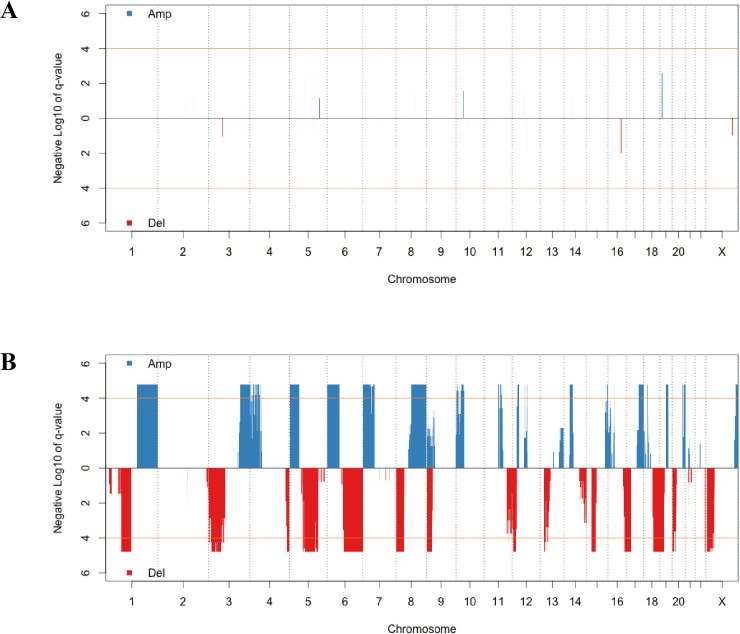
The distribution of recurrent amplification and deletion in paired normal tissues (A) and solid tumors (B). The blue and red blocks showed the amplification and deletion occurrences, respectively. The orange horizontal lines indicated the significant FDR cutoff of 10^−4^.

CNV has a critical role in cancer development and progression. A chromosomal segment can be deleted or amplified as a result of genomic rearrangements, such as deletions, duplications, insertions and translocations. The CNV in solid tumor tissues and the paired normal tissue were presented in Fig. 1. There were no significant segments in normal tissues from lung adenocarcinoma patients, while some regions were significantly amplified or deleted in the solid tumor sample. We annotated the regions with a FDR less than 10^−4^ as significant aberrant CNV segments.

**Table 2:** The pathway enrichment of genes involving in the recurrent amplification and deletion. Most of these pathways are associated with immune system.

Pathway	*P* Value	Benjamini
Deletion		
Regulation of autophagy	4.20E−07	8.00E−05
Natural killer cell mediated cytotoxicity	6.20E−06	5.90E−04
Amplification		
Systemic lupus erythematosus	5.30E−19	9.90E−17
Type I diabetes mellitus	3.80E−08	3.60E−06
Allograft rejection	2.20E−07	1.40E−05
Asthma	4.20E−06	2.00E−04
Graft-versus-host disease	6.10E−06	2.30E−04
Cell adhesion molecules (CAMs)	1.50E−04	4.60E−03
Antigen processing and presentation	5.10E−04	1.40E−02
Autoimmune thyroid disease	5.30E−04	1.20E−02
Intestinal immune network for IgA production	8.90E−04	1.80E−02
Viral myocarditis	1.40E−03	2.60E−02

**Figure 2:**
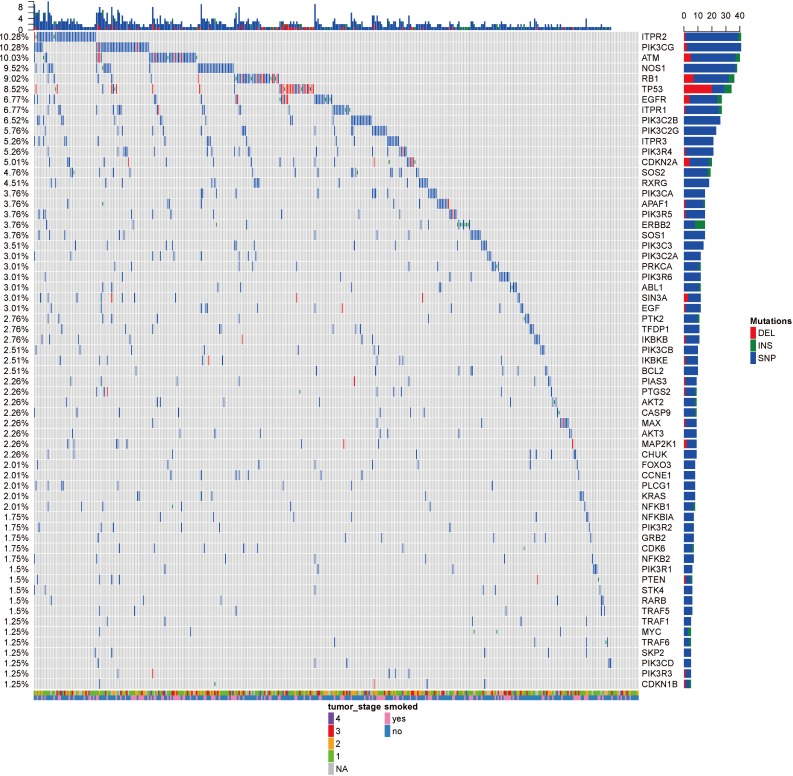
The OncoPrint of genes in small cell lung cancer signaling and non-small cell lung cancer signaling. SNPs were colored as blue, insertions as green and deletions as red. The upper barplot indicates the number of genetic mutation per patient, while the right barplot shows the number of genetic mutations per gene. The grades of lung adenocarcinoma and the history of smoking were added as annotations for the patients.

According to the significantly aberrant CNV segment, the genes which were fully located within the aberrant regions were identified by annotation from R package biomaRt (Durinck et al., 2009). The pathway enrichment was carried out for both the amplified genes and deleted genes (Table 2). The deleted genes were highly associated with pathways related with immunity, such as regulation of autophagy and natural killer cell mediated cytotoxicity. This suggested that the deletion of these genes led to the poorer immunity to disturbance like tumor and the progression of lung adenocarcinoma. The pathways enriched using amplified gene sets seemed to be irrelevant. However, most of these pathways were also associated with the immune system. For example, systemic lupus erythematosus is an autoimmune disease; allograft rejection was caused by foreign recognition by the recipient’s immune system; asthma occurred due to overactive immune system. Both the pathways enriched by the deleted or amplified genes were related to the immunity, which indicated the important roles that immunity system played on the homeostasis. Also, increasing the immunity activity or immunotherapy might be particularly effective and efficient for lung adenocarcinoma, compared with other cancer types.

### The recurrent mutations were identified

Analysis of 517 mutation annotated files, we highlighted 63 highly recurrent mutated genes which are associated with lung cancer signaling (Fig. 2). These genes are involved in important pathways related to cancer progression, including PI3K-Akt signaling pathway, MAPK signaling pathway, p53 signaling pathway and so forth. ITPR2, PIK3CG and ATM were commonly mutated (10.28%).

**Figure 3:**
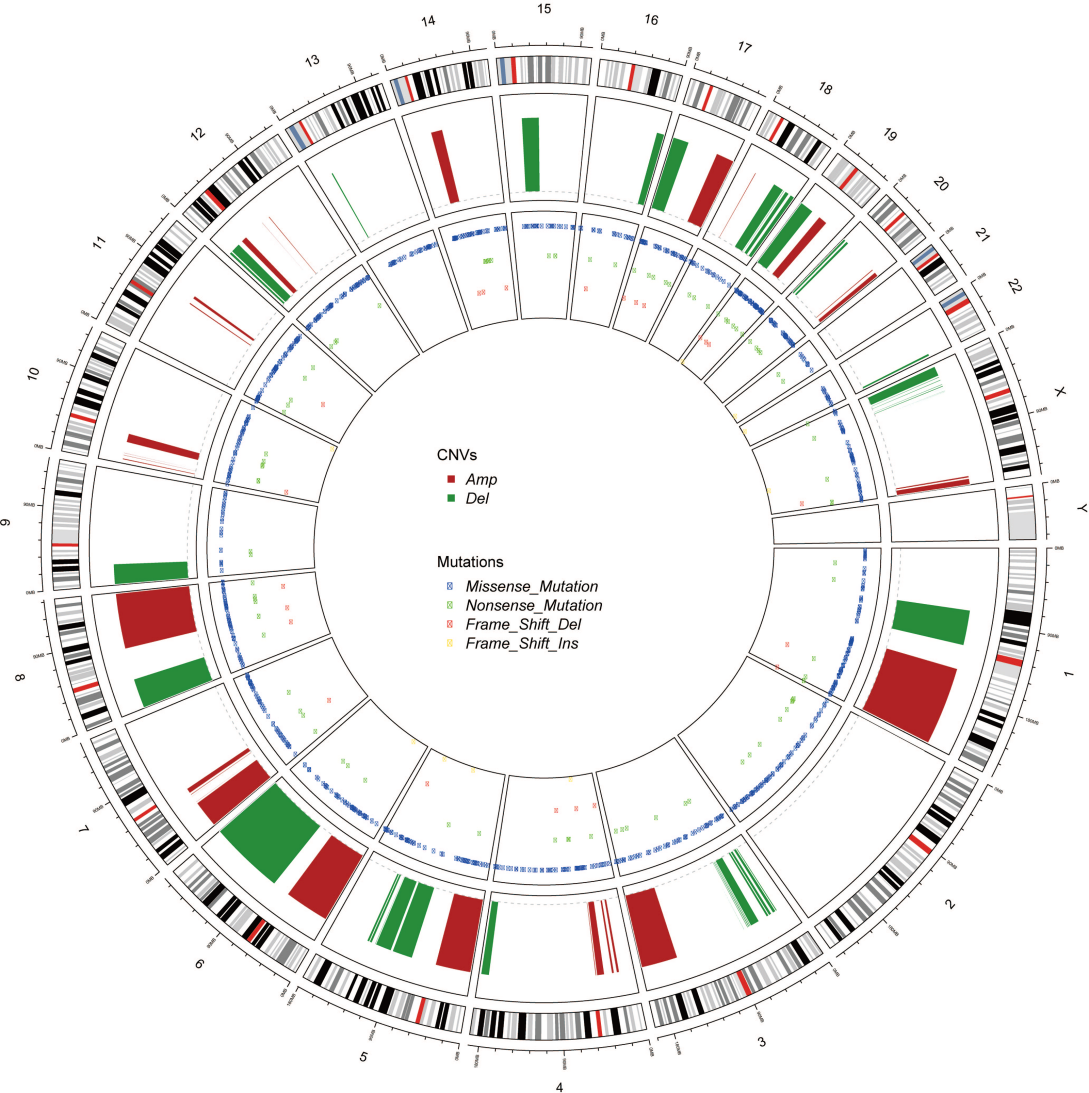
The Circos of the human genome showing chromosome structure, location of CNV (amplification and deletion) and the SNP (missense mutations, nonsense mutations, insertion and deletion) in lung adenocarcinoma. The most outer layer was the chromosome model, and the next two layers illustrated the CNV and SNP. Different genomic alterations were annotated as different color as noted in the center of the Circos.

**Table 3:** The genes involving in both the significantly aberrant CNV and the recurrent somatic mutations (Amp: copy number duplication; Del: copy number loss).

Symbol	Aberration	AberrantRegion	GeneRegion	MutFreq
RXRG	Amp	1:149879545–247813706	1:165400917–165445355	4.51%
PTGS2	Amp	1:149879545–247813706	1:186671791–186680427	2.26%
PIK3C2B	Amp	1:149879545–247813706	1:204422628–204494724	6.52%
IKBKE	Amp	1:149879545–247813706	1:206470476–206496889	2.51%
TRAF5	Amp	1:149879545–247813706	1:211326615–211374946	1.50%
AKT3	Amp	1:149879545–247813706	1:243488233–243851079	2.26%
RARB	Del	3:24385370–28226208	3:25174332–25597932	1.50%
PIK3CA	Amp	3:151906305–197538677	3:179148114–179240093	3.76%
SKP2	Amp	5:914233–45378456	5:36151989–36184319	1.25%
PIK3R1	Del	5:67686903–68318642	5:68215720–68301821	1.50%
ITPR3	Amp	6:1014281–57206485	6:33620365–33696574	5.26%
FOXO3	Del	6:77584009–170903919	6:108559835–108684774	2.01%
EGFR	Amp	7:49696467–56519285	7:55019021–55211628	6.77%
MYC	Amp	8:76221396–145232496	8:127735434–127741434	1.25%
PTK2	Amp	8:76221396–145232496	8:140657900–141002216	2.76%
CDKN2A	Del	9:789932–26141011	9:21967753–21995301	5.01%
CDKN1B	Del	12:7313760–19740546	12:12715058–12722371	1.25%
PIK3C2G	Del	12:7313760–19740546	12:18247614–18648416	5.76%
KRAS	Amp	12:24381497–32296427	12:25204789–25250936	2.01%
ITPR2	Amp	12:24381497–32296427	12:26336515–26833198	10.28%
NFKBIA	Amp	14:27325315–42171574	14:35401511–35404749	1.75%
TP53	Del	17:987221–21303007	17:7661779–7687550	8.52%
PIK3R6	Del	17:987221–21303007	17:8802723–8867677	3.01%
PIK3R5	Del	17:987221–21303007	17:8878911–8965712	3.76%
PRKCA	Amp	17:57842356–80917016	17:66302636–66810743	3.01%
GRB2	Amp	17:57842356–80917016	17:75318076–75405709	1.75%
BCL2	Del	18:62736694–63732867	18:63123346–63320128	2.51%
CCNE1	Amp	19:28240509–40248029	19:29811898–29824308	2.01%

The tumor stage and the smoke history were annotated in the bottom on the OncoPrint, which suggested that there were no relationship between the specific gene mutations with these to clinical features. Even though smoking is the leading cause of cancer, no specific gene was related to smoking. Ding et al. (2008) reported the different mutational profiles between smokers and non-smokers, but we only limited our scope within the lung cancer signaling, which might omit some patterns.

### Genomic variation analysis hinted PI3K protein family

Genomic alterations in cancer, including CNV and mutations, were represented as Circos (Krzywinski et al., 2009) (Fig. 3). Unlike the CNV which occurred unequally across all chromosomes, the mutations distributed normally. Also, the missense mutations were much more common than nonsense SNPs or frame shift mutations.

The intersections between the genes involving in the significantly aberrant CNV and the genes harboring recurrent somatic SNP were extracted (Table 3). This shortened list hinted the critical genes affecting the cancer initiation, progression and prognosis. Many famous cancer-related genes appeared in this list, which suggested the correctness of our analysis.

Another astonishing finding is that PI3K protein family acted as critical roles in the lung adenocarcinoma. The components of PI3K protein family include PIK3C2B, PIK3CA, PIK3R1 and so forth (Table 4). ITPR2 and ITPR3 were also the downstream of this protein family. These results suggested that we should pay attention on these genes and their protein products when we studied further guide diagnosis and treatment for lung adenocarcinoma.

**Table 4:** The genes with CNV or mutations involved in the PI3K signaling pathway.

Symbol	Aberration	Mut Freq	PI3K Class	Subunits
PIK3CA	Amp	3.76%	Class IA	p110*α*
PIK3R1	Del	1.50%	Class IA	p85*α*, p50*α*, p55*α*
PIK3R5	Del	3.76%	Class IB	p101
PIK3R6	Del	3.01%	Class IB	p87
PIK3C2B	Amp	6.52%	Class II	PI3K-C2*β*
PIK3C2G	Del	5.76%	Class II	PI3K-C2*γ*
ITPR1		6.77%	Downstream of lnc(1,4,5)P_3_	IP3R
ITPR2	Amp	10.28%		
ITPR3	Amp	5.26%		

## Discussion

Cancer Genome Atlas Research carried out a comprehensive analysis on molecular profiling of lung adenocarcinoma (Cancer Genome Atlas Research, 2014) which involved in 230 previously untreated lung adenocarcinoma patients. In this study, we utilized all the available data of TCGA lung adenocarcinoma which involved in 522 patients. In the meantime, their efforts were mainly put on comprehensive analysis, and this meant that they could not dig into specific CNV or SNP deeply. However, we aimed to figure out the gene alterations that might help personalized treatment. On the other hand, we used the level-3 data of TCGA lung adenocarcinoma, and all the data were pre-processed. It meant that we lost quite a bit of information. For example, the MAF file of mutations filtered out a lot of uncommon SNPs to protect the privacy of the patients. Considering the different samples and different analysis pipeline, there were some differences between our results and their results, like the rank of the most significantly differentially mutated genes, but there were no conflicted results.

As we have mentioned, we focused on the genomic alterations in lung adenocarcinoma. CNV is an important part of genomic changes, and it is a segment of DNA 1 kb or larger that is present in variable copy number and occur 100 to 10,000 times more frequently than point mutations in the human genome (Zhang et al., 2009). The importance of acquired chromosomal changes in tumorigenesis, including neuroblastoma, acute lymphoblastic leukemia, prostate cancer and breast cancer, has been established (Shlien et al., 2008). However, the role of constitutional CNVs in lung cancer has not yet been explored.

In this study, we found that the CNVs in the lung adenocarcinoma were mainly related with the immune system (Table 2). The deleted genes were directly associated with immunity containing pathways like regulation of autophagy and natural killer cell mediated cytotoxicity. Most of the pathways enriched by amplified genes were also associated with the immune system. For example, systemic lupus erythematosus is an autoimmune disease; allograft rejection was caused by foreign recognition by the recipient’s immune system; asthma occurred due to overactive immune systems. These results indicated increasing the immunity activity or immunotherapy might be particularly effective and efficient for lung adenocarcinoma, compared with other cancer types.

One explanation of this may be caused by the fact that smoking is the leading cause of lung adenocarcinoma, which caused inflammation and induced the cancer initiation. Prolonged exposure to environmental irritants, such as tobacco, can result in low-grade chronic inflammation that facilitated tumor development through induction of oncogenic mutations, genomic instability, early tumor promotion, and enhanced angiogenesis (Grivennikov, Greten & Karin, 2010). Such chronic inflammation is relatively seldom in other types of cancers.

Except for these enriched biological functions, PI3K signaling pathway seemed to be extremely critical in lung adenocarcinoma. Nine genes commonly mutated in lung adenocarcinoma solid tumor tissues, among which eight genes were also significantly aberrant CNVs, were involved in the PI3K signaling pathway (Table 4). This suggested the important roles of PI3K signaling pathway in the lung adenocarcinomas. One study reported the distinct patterns of genomic alterations in lung adenocarcinomas and squamous cell carcinomas (Campbell et al., 2016), and it seemed that PI3K3 had similar importance in squamous cell carcinomas.

PI3Ks are divided into Class I, Class II, and Class III according to their protein structures, lipid substrate specificity, *in vivo* distribution, mechanism of activation and function (Giudice & Squarize, 2013). Class I PI3Ks is usually categorized into two groups, Class IA and Class IB. Class IA PI3Ks consist of p110 catalytic subunits, which form protein complexes with p85 regulatory subunits. Class IB PI3Ks are formed by dimerization between the p110 δ and p101 or p87. Class II PI3Ks have three mammalian isoforms named PI3K-C2 α, PI3K-C2 β and PI3K-C2 γ (Table 4). According to our analysis, the p110 catalytic subunits, p101, PI3K-C2 β and PI3K-C2 γ showed copy number alterations, which suggested that both the Class I and II PI3Ks was affected in lung cancer while Class III might be normal-like.

**Figure 4:**
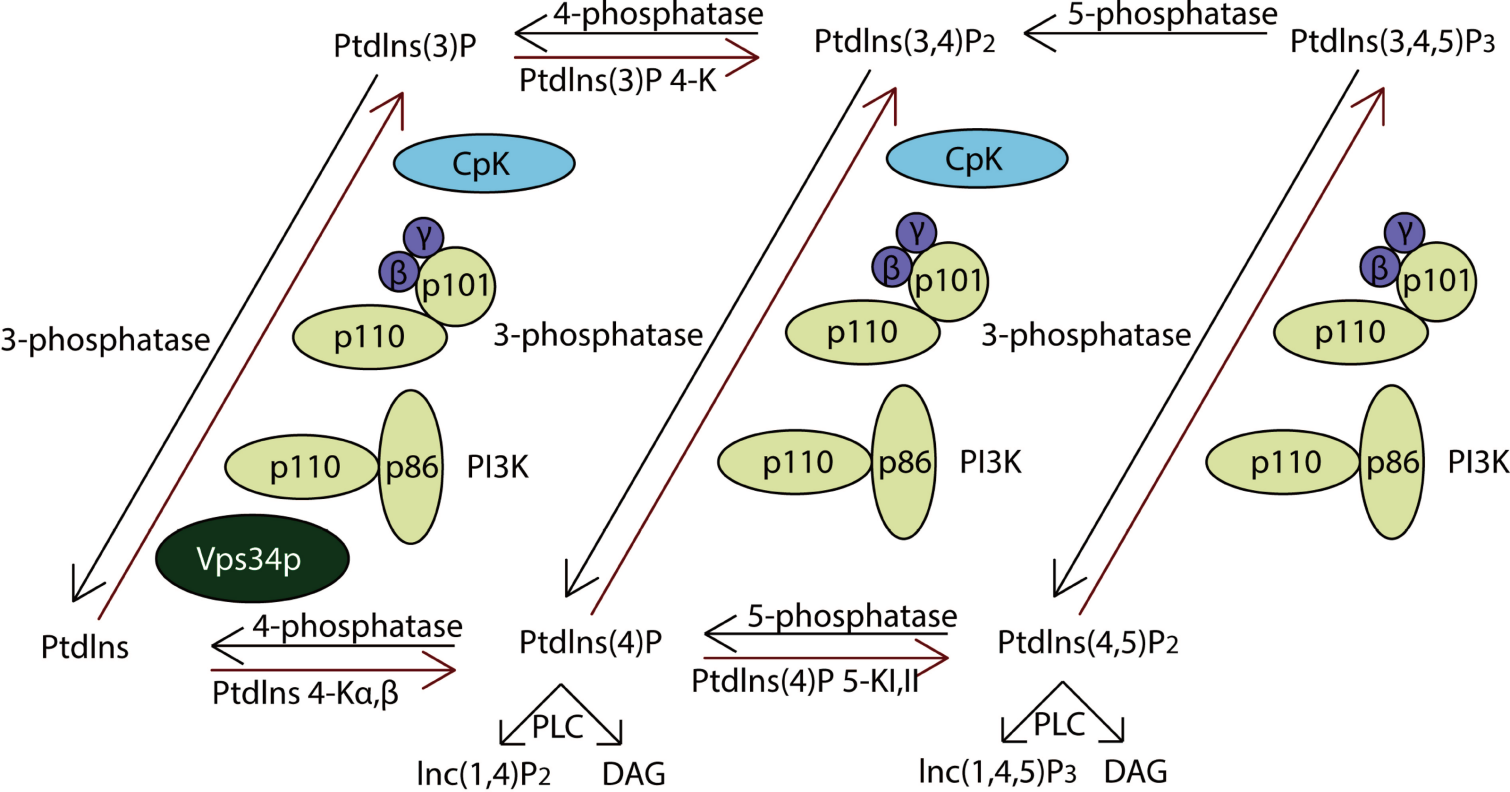
The pathway of PI3K protein family. PI3Ks works as intracellular lipid kinases that phosphorylate Ptdlns and phosphoinositides and the graph showed its metabolism. The components directly involving in the genomic alterations were annotated by the red stars.

PI3Ks work as intracellular lipid kinases that phosphorylate Ptdlns and phosphoinositides (Fig. 4). The substrates for class I PI3Ks are Ptdlns, Ptdlns(4)P, and Ptdlns(3,4)P_2_ but they primarily catalyzes the conversion of Ptdlns(3,4)P_2_ to Ptdlns(3,4,5)P_3_, which activates many downstream signaling proteins, including AKT (Sasaki et al., 2009). The class II PI3Ks preferentially phosphorylate PtdIns to generate PtdIns(3)P but can also phosphorylate PtdIns(4)P to yield PtdIns(3,4)P_2_.

Activation of class IA PI3Ks predominantly generates PtdIns(3,4,5)P3, which is a crucial activator of Akt. In further, they play critical roles in cell survival, metabolism and cancer progression. IP3Rs (inositol 1,4,5-trisphosphate receptors), encoded by ITPR1, ITPR2 and ITPR3, were the downstream of PI3K pathway. They are control cell survival, adaptation and death processes through regulating Ca^2+^-signaling and entry (Stephens et al., 2004). As a result, the PI3K pathway impacts most cellular functions involved in tumor behavior, including cell growth, local invasion, metastasis, survival, and resistance to therapy.

However, there are few studies indicating the roles of class II PI3Ks on cancer. Our results suggested that they might be also critical for lung adenocarcinoma, which gave a direction for further studies.

## Additional Information and Declarations

### Data Availability

The following information was supplied regarding data availability: The raw data is available at https://gdc-portal.nci.nih.gov/projects/TCGA-LUAD.
